# Work participation, social roles, and empowerment of Q-fever fatigue syndrome patients ≥10 years after infection

**DOI:** 10.1371/journal.pone.0302573

**Published:** 2024-04-30

**Authors:** I. M. Brus, A. S. J. Teng, S. C. M. Heemskerk, S. Polinder, P. Tieleman, E. Hartman, B. Dollekens, J. A. Haagsma, I. Spronk

**Affiliations:** 1 Department of Public Health, Erasmus MC, University Medical Center Rotterdam, Rotterdam, the Netherlands; 2 Q-support, ‘s Hertogenbosch, the Netherlands; West University of Timisoara: Universitatea de Vest din Timisoara, ROMANIA

## Abstract

**Objective:**

To determine work participation, social roles, and empowerment of QFS patients ≥10-year after infection.

**Methods:**

QFS patients ≥10-year after acute infection, who were of working age, participated in a cross-sectional survey study. Work participation, fulfilment of social roles, and empowerment outcomes were studied for the total population, as well as for subgroups based on employment type and current work status. Associations between empowerment, work and social roles were examined.

**Results:**

291 participants were included. Of the 250 participants who had paid work before Q-fever, 80.4% stopped working or worked less hours due to QFS. For each social role, more than half of the participants (56.6–87.8%) spent less time on the role compared to before Q-fever. The median empowerment score was 41.0 (IQR: 37.0–44.0) out of 60. A higher empowerment score was significantly associated with lower odds of performing all social roles less due to QFS (OR = 0.871–0.933; p<0.001–0.026), except for parenting and informal care provision (p = 0.070–0.460). No associations were found between empowerment and current work status.

**Conclusion:**

Work participation and fulfilment of social roles is generally low in QFS patients. Many of the participants stopped working or are working less hours due to QFS, and most spent less time on social roles compared to before Q-fever. Minor variation was seen in total empowerment scores of participants; however, these slight differences were associated with the fulfilment of social roles, but not work participation. This new insight should be further explored in future studies.

## Introduction

Q-fever fatigue syndrome (QFS), which is a sequela of the zoonotic disease Q-fever, can have a large impact on patients’ lives [1, 2]. Between 2007 and 2010, a large Q-fever outbreak took place in the Netherlands, with over 50,000 individuals being infected, which posed a major public health problem for the authorities [3, 4]. Knowledge on the long-term impact of Q-fever was scarce and available care for those suffering from these long-term consequences was limited. Recent research shows that patients are unsatisfied with the quality of care for QFS, even years after the outbreak [2]. However, attention for these long-term consequences is crucial as about one out of five individuals infected with Q-fever develop QFS and have to live with chronic fatigue symptoms [5]. Moreover, a large proportion of QFS patients face additional short- and long-term physical and/or psychological problems impacting their health status and health-related quality of life [2, 5–9]. Also, their ability to participate in daily life can be (strongly) impacted [2, 10]. Work and social participation can improve patients’ health, quality of life, well-being, and life satisfaction, and is thus important for patients [11]. Furthermore, work has personal, social, and economic benefits for patients and can be protective factor for mental health, as well as promote recovery and inclusion in the community [12]. Prolonged sick leave or not returning to work may result in inactivity and isolation, and decreased personal income [13].

Previous research in Q-fever patients, including QFS patients, showed that during the acute infection, over 90% of patients were on sick leave, with more than half of patients resuming work within one month after infection, and most resuming work within one year [14]. However, about 30% of patients did not resume work fully, or were unable to function at pre-infection level [14, 15]. Four years post Q-fever, almost half of QFS patients had stopped working. In addition, the average number of working hours of patients who remained or resumed working decreased considerably; from 35h/week prior to 22h/week. Their work ability was significantly lower compared to the general population [10]. Another study showed that, nine years post Q-fever, slightly more than half of patients experienced problems with the social domain ‘work’ due to Q-fever [2]. About one third of patients stopped working and another third worked less hours after being infected compared to before Q-fever, with an average work time reduction of 50% [2].

Few studies have investigated the impact on QFS on social participation. One previous study compared social participation between patients with past acute Q-fever and QFS and found that patients with QFS had a significantly lower level of social participation than those with past acute Q-fever [10]. In addition, the level of social participation of QFS patients showed no improvement over time up to 8 years after infection. A study among patients with QFS, chronic Q-fever, and QFS-like disease showed that their ability to fulfil social roles, such as hobbies, leisure and sport activities, was strongly impacted [2].

To the best of our knowledge, no studies have been conducted on the impact of QFS on work participation and the fulfilment of social roles ≥10 years after Q-fever. In addition, it is not yet known what factors are associated with long-term work participation and social roles. A factor of interest is patients’ empowerment, which is defined as a process in which individuals can influence situations that are important to them [16–18]. Empowerment might be an important factor in social and work participation of QFS patients. Earlier studies showed that higher levels of empowerment were associated with enhanced health status and quality of life as well as improved social and work participation in patients with chronic diseases [19–22]. Besides, interventions aiming at empowering patients were shown to be effective in improving the empowerment of patients [19, 23–25]. Therefore, this study determined work participation, social roles, and empowerment of QFS patients ≥10 years after Q-fever, and examined the association of empowerment with work participation and fulfilment of social roles.

## Methods

### Study design and participants

Approval of this survey study was provided by the Medical Ethics Review Board of Erasmus MC (MEC-2021-1606). The study was conducted following the principles of the Declaration of Helsinki, reported in line with the STROBE guidelines [26], and conducted in collaboration with Q-support, the Q-fever expertise centre in the Netherlands [27]. The start date of the recruitment period was 22 September 2021 and the end date 2 December 2021.

QFS patients who were registered at Q-support in September 2021, received an e-mail invitation to participate in this study. Study information and a link to the online survey was included in the invitation. Participation was voluntary, and all participants provided online written informed consent before answering the survey questions. If preferred, patients could request a paper version of the survey by mail. In case patients did not respond to the survey within two weeks, a reminder was sent. A second reminder was sent when a patient did not respond within two weeks after the first reminder; and a third reminder was sent two weeks after no response to the second reminder.

Patients ≥10-year post Q-fever were selected in the present study. In case year of acute infection was unknown, the year of the QFS diagnosis was used. If both were unknown, patients were excluded. Besides, we only included patients in the working age; patients aged ≥18 years old at time of Q-fever and <67 years old (Dutch retirement age in 2021) at time of survey.

### Survey

#### Sociodemographic and medical characteristics

The survey was developed in collaboration with QFS patients and QFS healthcare providers. Questions on sociodemographic characteristics included age, gender, level of education, and living situation. Level of education was categorized into low, middle and high level of education according to the International Standard Classification of Education [28]. Questions on medical characteristics included self-reported year of acute infection, hospitalization during the acute infection, use of antibiotics during the acute infection, and coexisting chronic diseases at the time of the survey [29].

#### Work participation

Seven items on work were included in the survey. These items covered pre-infection employment type, which was recoded into three categories: i) wage-employment, ii) self-employment, and iii) no employment (e.g., volunteer work, job seeker). If participants were both wage-employed and self-employed, they were classified as wage-employed; if participants were both wage-employed and did volunteer work, they were classified as wage-employed; if participants were both self-employed and did volunteer work, they were classified as self-employed. Participants who were wage-employed or self-employed pre-infection were asked about their current work status. Answer options were categorised into: ‘currently working less hours due to QFS’, ‘stopped working due to QFS’, and ‘other work status’. Participants were asked if they were incapacitated to work due to QFS or another reason. If participants were incapacitated to work due to QFS, they indicated who determined their incapacity, if they were able to resume work, their current percentage of incapacity to work, and if they received disability benefits for their incapacity to work. Work related outcomes were studied for and compared between wage-employed and self-employed participants.

#### Social roles

Participants were asked to estimate their fulfilment of nine social roles compared to before Q-fever. If a specific role was applicable to them, they indicated whether they spend more, the same or less time on relationship with a partner, household activities, sport, social contacts, hobbies, volunteer work, informal care provision, parenting, and traveling due to QFS.

#### Empowerment

Empowerment was assessed with a shortened version of the Netherlands Empowerment List [16]. Twelve items were included which were scored on a 5-point Likert scale ranging from 1 (‘strongly disagree’) to 5 (‘strongly agree’). In line with the original version, a total sum score was calculated, ranging from 12 to 60. Higher scores indicate more empowerment.

### Statistical analysis

Sociodemographic and medical characteristics as well as outcomes were described using descriptive statistics. Mean (SD) was used to report continuous data if normally distributed, otherwise, median (IQR) was reported. Categorical data were reported as numbers and percentages. ANOVA or Kruskal-Wallis tests (continuous variables) and chi-square tests or Fisher’s exact tests (n<5) (categorical variables) were performed to determine differences in sociodemographic and medical characteristics between the three subgroups based on pre-infection employment type. Post-hoc Bonferroni tests were used for multiple comparisons if results were statistically significant (p<0.05). Work related outcomes were compared between wage-employed and self-employed participants. To evaluate differences between these two subgroups Mann Whitney U tests (continuous variables), and chi-square tests or Fisher’s exact tests (n<5) (categorical variables) were done. Differences in empowerment sum scores between participants who performed social roles less than before infection and those who performed roles as much or more than before were studied using Mann Whitney U tests. Multinomial logistic regression analyses were performed to identify determinants associated with current work status. Logistic regression analyses were performed to identify determinants of performing social roles less than before Q-fever. Sociodemographic characteristics, medical characteristics, and empowerment sum score were included as independent variables. Variables with a p-value <0.10 in univariate analyses were included in multivariate regression analyses after checking for collinearity (>0.8 or <-0.8). A p-value of <0.05 was considered statistically significant. All analyses were performed using IBM SPSS version 28.

## Results

### Patient characteristics

In total, 377 of the 671 eligible patients (response rate: 56.2%) completed the survey. Of these patients, 291 met our inclusion criteria and were included in the present study (S1 Fig). Slightly more than half were females (54.3%) and the median age at time of study was 54.0 (IQR: 48.0–61.0) (Table 1), which was comparable to these characteristics of the eligible population: 53.0% females; median age 52.0 years old. Many participants were married or lived together with a partner (71.8%) and had a coexisting chronic disease (62.9%). The median time since Q-fever at time of study was 12 years. The majority of patients received antibiotics during the acute phase of the infection (64.9%). No information was available about why the remaining patients did not receive antibiotics; they might not have consulted a physician during the acute phase. A total of 17.2% was hospitalized during the acute phase.

**Table 1:** Characteristics of study sample.

		Total sample (n = 291)
Age at time of study, median (IQR)		54.0 (48.0–61.0)
**Gender**		
	Male	133 (45.7%)
	Female	158 (54.3%)
**Level of education**		
	Low	77 (26.5%)
	Middle	128 (44.0%)
	High	86 (29.6%)
**Household composition**		
	Married or living with partner, with or without children at home	209 (71.8%)
	Living alone or one-parent household with or without children living at home	82 (28.2%)
**Coexisting chronic disease**		
No coexisting chronic disease		108 (37.1%)
≥1 coexisting chronic disease		183 (62.9%)
Years since Q-fever, median (IQR)		12.0 (12.0–13.0)
	Before 2007	17 (5.8%)
	Between 2007–2011	274 (94.2%)
**Antibiotics during the acute phase of the infection**		
Yes		189 (64.9%)
No		84 (28.9%)
Not sure		18 (6.2%)
**Hospitalization during the acute phase of the infection**		
Yes		50 (17.2%)
No		241 (82.8%)

Of the 290 participants who were not retired at time of filling in the survey, most were wage-employed before Q-fever (74.1%). The remaining participants were fully self-employed (12.1%) or had no employment (13.8%). These three subgroups differed in age, gender, level of education, and number of coexisting chronic diseases (p = 0.002–0.025; S1 Table). Self-employed participants were older (median: 59 years; IQR: 55.0–61.0), were more often male (65.7%), and more often had a coexisting chronic disease (71.4%).

### Work participation

Of the 250 participants who had paid work before Q-fever and were not retired at the time of the survey, almost half (48.0%) had completely stopped working at time of study (≥10 years post infection) (Table 2). Of the 120 participants who permanently stopped working, more than half (n = 65; 54.2%) never resumed work since infection. A third (32.4%) of the participants who had paid work before Q-fever were working less hours due to QFS at time of study. Another 6.4% had worked less between infection and time of study but had resumed their pre-infection working hours at time of study. Although there were no statistically significant differences between participants who were wage-employed and self-employed (p>0.05); a trend was observed. A higher proportion of wage-employed participants completely stopped working (49.8% vs 37.1%), whereas a higher proportion of self-employed participants worked less hours (48.6% vs 29.8%) due to QFS.

**Table 2:** Work related outcomes for participants who were employed before Q-fever.

	Total subsample (n = 250)	Wage-employed (n = 215)	Self-employed (n = 35)	P-value for difference
**Current work status**				0.060
Permanently stopped working due to QFS	120 (48.0%)	107 (49.8%)	13 (37.1%)	
Work less hours due to QFS	81 (32.4%)	64 (29.8%)	17 (48.6%)	
Working hours have changed, but not due to QFS	12 (4.8%)	10 (4.7%)	2 (5.7%)	
Currently the same working hours as before infection, but have worked less before due to QFS	16 (6.4%)	13 (6.0%)	3 (8.6%)	
Nothing has changed	21 (8.4%)	21 (9.8%)	0 (0.0%)	
**Periods unable to work since Q-fever infection**				0.470
No	35 (14.0%)	32 (14.9%)	3 (8.6%)	
Yes	150 (60.0%)	126 (58.6%)	24 (68.6%)	
Never worked since infection	65 (26.0%)	57 (26.5%)	8 (22.9%)	
**Incapacitated**				0.496
No	110 (44.0%)	96 (44.7%)	14 (40.0%)	
(Partially) incapacitated due to QFS	132 (52.8%)	113 (52.6%)	19 (54.3%)	
(Partially) incapacitated not due to QFS	8 (3.2%)	6 (2.8%)	2 (5.7%)	
Resumed to work after period(s) of incapacity to work due to QFS*				0.489
Yes	20 (15.2%)	16 (14.2%)	4 (21.1%)	
No	112 (84.8%)	97 (85.8%)	15 (78.9%)	
Percentage of current incapacity to work*				0.004
**Median (IQR)**	100.0 (75.0–100.0)	100.0 (79.0–100.0)	75 (53.0–100.0)	
Receiving disability benefit for incapacity to work due to QFS*				<0.001
Full benefit	79 (59.8%)	75 (66.4%)	4 (21.1%)	
Partial benefit	25 (18.9%)	21 (18.6%)	4 (21.1%)	
State pension	4 (3.0%)	4 (3.5%)	0 (0.0%)	
No	24 (18.2%)	13 (11.5%)	11 (57.9%)	

*Only for participants who were incapacitated due to QFS.

Over half of the participants (52.8%) was (partially) incapacitated due to QFS; only 15.2% of them resumed to work after a period of incapacity. The median percentage of incapacitation at time of study was 100.0% (IQR: 75.0–100.0); most of the incapacitated participants (69.7%) were incapacitated for ≥80%. No differences on incapacitation outcomes were found between wage-employed and self-employed participants, apart from the median percentage of incapacitation; that was statistically significantly higher in wage-employed compared to self-employed participants (median 100.0% vs 75.0%; p = 0.004). The majority of participants who were incapacitated to work due to QFS received a (partial) disability benefit (78.7%). This proportion was higher in wage-employed compared to self-employed participants (85.0% vs. 42.2%; p<0.001). Of those receiving a disability benefit, the vast majority of wage-employed participants (96.9%) received a disability benefit from the Employee Insurance Agency (UWV), whereas the majority of the self-employed participants received it from an insurance company (62.5%; p<0.001).

### Social roles

**Fig 1:**
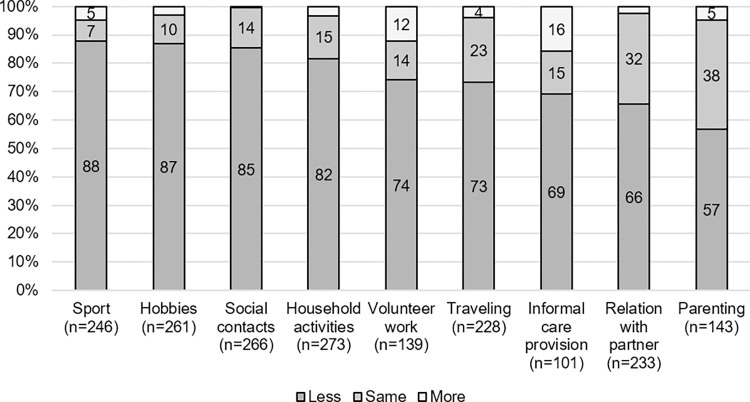
Fulfilment of social roles. Fulfilment of social roles in comparison to before Q-fever for total sample of patients, sorted by percentage ‘less than before Q-fever’*. *Percentages ≤3 are not presented as numbers in the figure; number of patients per role differed as not all roles were relevant for all patients.

Almost all participants (97.8%) spent less time on at least one of the social roles they had before the infection. Almost half (42.4%) spent less time on all of their social roles. If a specific role was applicable, the majority (56.6–87.8%) spent less time on each social role studied compared to before Q-fever (Fig 1). Many participants spent less time on sport (87.8%) and hobbies (87.0%). Time spent on some roles increased for a part of the participants, with the proportion being largest for informal care provision (15.8%), and volunteer work (12.2%).

### Empowerment

**Fig 2:**
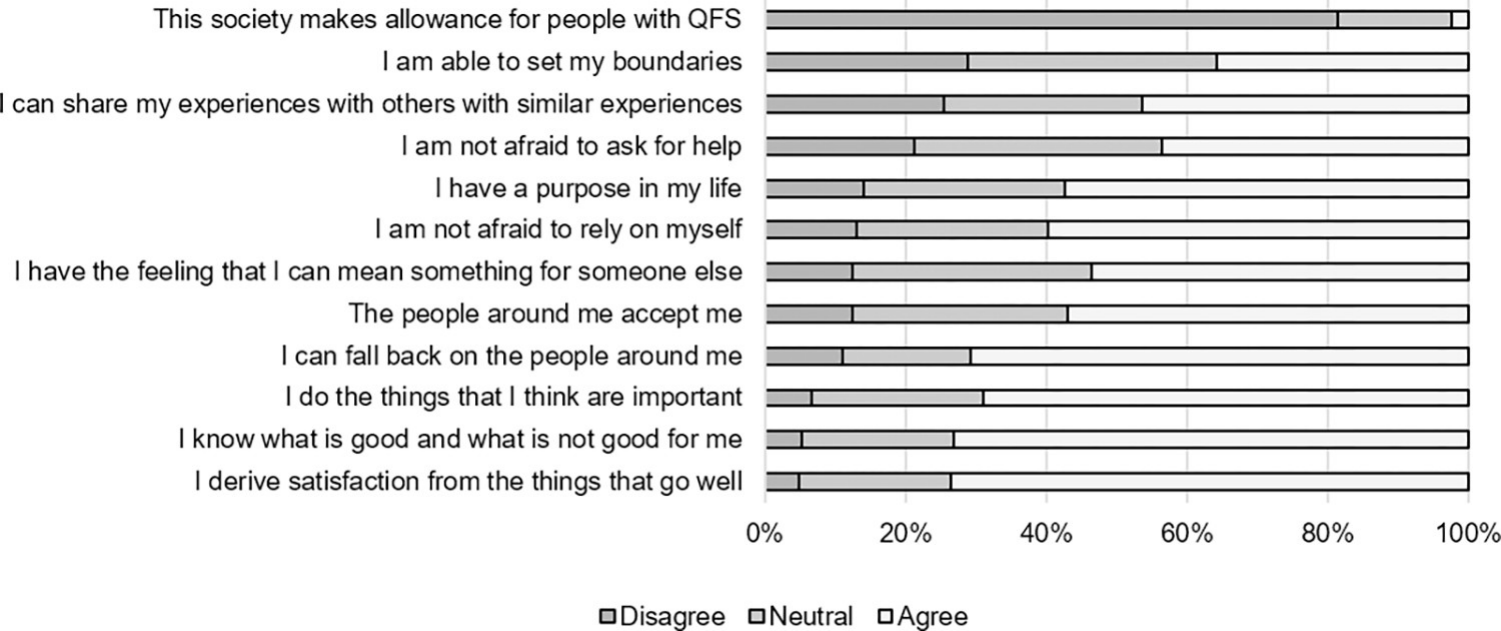
Empowerment of QFS patients ≥10 years post Q-fever.

The mean empowerment sum score was 40.4 (SD: 5.5) and the median score was 41.0 (IQR: 37.0–44.0) out of 60. The level of agreement of participants on the different empowerment statements is presented in Fig 2. Concerning external factors: many participants reported that society makes no allowance for people with QFS (81%), however, slightly more than half reported that the people around them accept them (57%). Many participants (>70%) indicated that they felt empowered on some internal factors: deriving satisfaction from things that go well, and knowing what is good for them and what is not. However, empowerment was not experienced on all aspects, as only 44% of the participants was not afraid to ask for help, and only 36% was able to set their own boundaries.

### Empowerment, work and social roles

When comparing the empowerment sum score among subgroups based on the current work status of participants, no statistically significant differences were found (median score: 41.0–42.0; p = 0.213). When comparing the empowerment sum score among subgroups based on social role performance, significant differences in the empowerment sum score were found between participants who fulfilled the role relationship with partner less than before QFS and those who performed this role as much or more than before, with those performing the role as much or more having higher empowerment scores (p<0.001) (Table 3). This is also the case for the roles household activities (p = 0.035), hobbies (p<0.001), sport (p = 0.006), social contacts (p = 0.005), volunteer work (p<0.001), and travelling (p = 0.006). No significant difference was found for the roles parenting or informal care provision.

**Table 3:** Empowerment score for subgroups based on social role performance compared to before Q-fever.

	N (%)*	Empowerment sum score, median (IQR)	Mean rank	P-value for difference
**Relationship with partner**				
Less	153 (65.7)	40.0 (7.0)	105.54	<0.001
As much or more	80 (34.3)	42.0 (7.0)	138.93	
**Household activities**				
Less	223 (81.7)	41.0 (7.0)	132.25	0.035
As much or more	50 (18.3)	42.5 (8.0)	158.20	
**Parenting**				
Less	81 (56.6)	40.0 (7.5)	70.41	0.600
As much or more	62 (43.4)	41.0 (8.0)	74.07	
**Hobbies**				
Less	227 (87.0)	41.0 (8.0)	125.11	0.001
As much or more	34 (13.0)	42.5 (5.5)	170.34	
**Sports**				
Less	216 (87.8)	41.0 (7.8)	118.87	0.006
As much or more	30 (12.2)	42.5 (6.3)	156.87	
**Social contacts**				
Less	227 (85.3)	41.0 (7.0)	128.08	0.005
As much or more	39 (14.7)	43.0 (7.0)	165.03	
**Volunteer work**				
Less	103 (74.1)	41.0 (8.0)	63.27	<0.001
As much or more	36 (25.9)	44.0 (4.8)	89.26	
**Travelling**				
Less	167 (73.2)	41.0 (8.0)	107.24	0.006
As much or more	61 (26.8)	43.0 (6.5)	134.39	
**Informal care provision**				
Less	70 (69.3)	40.0 (6.3)	47.44	0.065
As much or more	31 (30.7)	42.0 (8.0)	59.05	

*Number of patients per role differed as not all roles were relevant for all patients.

### Determinants of current work status and social roles

Multinomial logistic regression analyses on current work status are presented in Table 4. None of determinants studied was associated with participants’ current work status. Univariate and multivariate logistic regression analyses on performing social roles less than before Q-fever are presented in Tables 5–7 and S2–S4 Tables. The empowerment sum score was significantly associated with lower odds of performing a role less than before QVS for all social roles (OR = 0.871–0.933, p<0.001–0.026), except for parenting and informal care provision, in both univariate and multivariate analyses. In addition, male gender was significantly associated with lower odds of performing the roles household activities (OR = 0.198, p<0.001), volunteer work (OR = 0.313, p = 0.011), and informal care provision (OR = 0.369, p = 0.027) less than before QVS in univariate and multivariate analyses. Not being married or living with a partner was associated with higher odds of performing the roles volunteer work, travelling, and relationship with partner less than before in univariate analyses, and only travelling (OR = 2.711, p = 0.013) and relationship with partner (OR = 4.185, p = 0.003) in multivariate analyses. Lastly, a lower education level was associated with higher odds of performing household activities less than before in univariate and multivariate analyses (OR = 2.536, p = 0.041).

**Table 4:** Univariate multinomial logistic regression analyses on current work status.

	Less hours work due to QFS			Stopped working due to QFS		
	Base outcome: other working status					
	*OR*	*95% CI*	*p-value*	*OR*	*95% CI*	*p-value*
**Gender**						
Male	0.525	0.256–1.078	0.079	0.667	0.340–1.307	0.238
Female (ref)						
**Age** (continuous)	0.973	0.936–1.011	0.156	1.021	0.983–1.060	0.279
**Level of education**						
Low	1.059	0.455–2.465	0.895	1.101	0.499–2.427	0.812
Middle (ref)						
High	1.727	0.765–3.901	0.189	1.929	0.895–4.157	0.093
**Married/living with partner**						
Yes (ref)						
No	1.762	0.739–4.205	0.201	1.830	0.803–4.169	0.150
**Comorbidity**						
None	0.965	0.471–1.977	0.921	0.800	0.407–1.572	0.518
≥1 (ref)						
**Hospitalization**						
No (ref)						
Yes	1.181	0.480–2.902	0.718	0.784	0.325–1.890	0.588
**Empowerment sum score (continuous)**	0.946	0.884–1.013	0.111	0.956	0.896–1.019	0.165

**Note**. p-values printed in bold indicate statistically significant values (p<0.05).

**Table 5:** Multivariate logistic regression analyses for performing a specific social role less than before Q-fever for the roles: Relationship with partner, household activities, and parenting.

	Relationship with partner			Household activities			Parenting		
	N = 233			N = 273			N = 143		
	*OR*	*95% CI*	*p-value*	*OR*	*95% CI*	*p-value*	*OR*	*95% CI*	*p-value*
**Gender**									
Male				0.198	0.097–0.407	**<0.001**			
Female (ref)									
**Age** (continuous)									
**Level of education**									
Low				2.536	1.037–6.203	**0.041**			
Middle (ref)									
High				1.538	0.720–3.284	0.266			
**Married/living with partner**									
Yes (ref)	4.185	1.642–10.669	**0.003**						
No									
**Paid work before Q-fever**									
Yes (ref)									
No									
**Comorbidity**									
None									
≥1 (ref)									
**Hospitalization**									
No (ref)									
Yes									
Empowerment sum score (continuous)	0.898	0.846–0.954	**<0.001**	0.924	0.865–0.986	**0.018**			

**Note**. This table presents the odds ratio (OR) for performing a specific role **less** than before Q-fever.

**Note**. p-values printed in bold indicate statistically significant values (p<0.05).

**Table 6:** Multivariate logistic regression analyses for performing a specific social role less than before Q-fever for the roles: Hobbies, sports, and social contacts.

	Hobbies			Sports			Social contacts		
	N = 261			N = 246			N = 266		
	*OR*	*95% CI*	*p-value*	*OR*	*95% CI*	*p-value*	*OR*	*95% CI*	*p-value*
**Gender**									
Male									
Female (ref)									
**Age** (continuous)									
**Level of education**									
Low									
Middle (ref)									
High									
**Married/living with partner**									
Yes (ref)									
No									
**Paid work before Q-fever**									
Yes (ref)									
No									
**Comorbidity**									
None									
≥1 (ref)									
**Hospitalization**									
No (ref)									
Yes									
Empowerment sum score (continuous)	0.871	0.803–0.944	**<0.001**	0.888	0.817–0.964	**0.005**	0.904	0.841–0.971	**0.006**

**Note**. This table presents the odds ratio (OR) for performing a specific role **less** than before Q-fever.

**Note**. p-values printed in bold indicate statistically significant values (p<0.05).

**Table 7:** Multivariate logistic regression analyses for performing a specific social role less than before Q-fever for the roles: Volunteer work, travelling, and informal care provision.

	Volunteer work			Travelling			Informal care provision		
	N = 139			N = 228			N = 101		
	*OR*	*95% CI*	*p-value*	*OR*	*95% CI*	*p-value*	*OR*	*95% CI*	*p-value*
**Gender**									
Male	0.313	0.128–0.765	**0.011**				0.369	0.152–0.895	**0.027**
Female (ref)									
**Age** (continuous)	0.961	0.914–1.010	0.115						
**Level of education**									
Low	4.571	0.911–22.941	0.065						
Middle (ref)									
High	0.800	0.321–1.993	0.632						
**Married/living with partner**									
Yes (ref)									
No	2.449	0.863–6.952	0.093	2.711	1.235–5.950	**0.013**			
**Paid work before Q-fever**									
Yes (ref)									
No									
**Comorbidity**									
None									
≥1 (ref)									
**Hospitalization**									
No (ref)									
Yes									
Empowerment sum score (continuous)	0.902	0.826–0.984	**0.020**	0.933	0.878–0.992	**0.026**	0.923	0.847–1.007	0.070

**Note**. This table presents the odds ratio (OR) for performing a specific role **less** than before Q-fever.

**Note**. p-values printed in bold indicate statistically significant values (p<0.05).

## Discussion

Work participation, fulfilment of social roles, and empowerment of QFS patients ≥10 years after Q-fever were determined in this study. Both work participation and social roles were highly impacted. The vast majority of the participants stopped working or worked less hours due to QFS ≥10 years after the infection. Over half of the participants were (partially) incapacitated due to QFS, with two thirds of them being incapacitated for at least 80%. The impact of QFS on the fulfilment of social roles was also substantial; most participants spent less time on most social roles compared to before Q-fever. Sports and hobbies appeared to be most affected, whereas parenting, relationship with partner and informal care provision were least affected, although the majority of participants still performed these roles less. Little variation was seen in total empowerment scores of participants; however, these small differences were related with social role fulfilment, but not work participation. Participants with lower empowerment scores had significantly higher odds of fulfilling all social roles less than before infection, except for parenting and informal care provision. None of the other determinants studied showed such a consistent trend in the association with social roles. Female gender was associated with less household activities, volunteer work, and informal care provision; lower education level with less household activities; and not being married/living with a partner only with travelling less, all compared to the situation before Q-fever. None of the determinants studied were associated with current work status.

In line with earlier studies, our study showed that the impact of QFS on work participation is high [2, 10]. A previous study reported that four years post Q-fever, 47% of the QFS patients had stopped working [[10], which is almost identical to the 48% that has stopped working in our study ≥10 years after Q-fever. Even though this is not the same population, it seems to indicate that many patients stop working within the first four years post infection. The working capabilities of many participants that remained working were impacted by QFS as two thirds of them worked less hours due to QFS compared to before the infection. This not only highly impacts the patients themselves, both socially and financially, but also comes with high societal costs [14, 30, 31]. A trend was seen in our results that wage-employed participants more often stopped working completely, whereas a higher proportion of self-employed participants worked less hours due to QFS. However, wage-employed participants were not statistically significantly more often incapacitated, though if incapacitated, their median percentage of incapacity to work was significantly higher. It is thus possible that self-employed participants experience a lower impact of QFS on their ability to work than employees. However, the observed differences might be, at least partially, caused by the different (financial) regulations and legislation between these two groups in the Netherlands [32, 33]. The financial support system for wage-employed people in the Netherlands is relatively strong: for example, employers are obliged to pay at least 70% of the previous wage for a maximum of two years, as well as help the employee in their return-to-work process, including job adaptations and/or getting a new job. In contrast, self-employed workers must organize their own disability insurance, which is not compulsory and is considered quite expensive [34]. Due to these differences, self-employed workers might experience more financial pressure to keep working, at least a few hours a week, although the impact of QFS on their working abilities might be similar to wage-employed patients. Our results might also be partially explained by findings of an earlier study that showed that self-employment leads to an increase in working ability over time [35]. Self-employed participants might have been able to cope better with QFS, due to a higher ability of adjusting their tasks to the impact of QFS on their work. For future research, distinguishing self-employed patients with and without disability insurance would be relevant, as the financial support they receive in case of sick leave differs considerably [32, 33].

To the best of our knowledge, only one other study assessed the impact of QFS on the fulfilment of social roles [2]. Nine years after Q-fever, QFS negatively impacted all eleven social roles studied, with patients reporting the greatest impact on work and exercise, and the least on relationship and self-care [2]. This is in line with our results, which show that the roles sport and hobbies were most affected, and relationship with partner and parenting were least affected. This distinction is probably caused by the nature of these roles and the obligation or necessity to fulfil them. Reducing time spent on sport and hobbies is likely to be easier than reducing time spent on for example parenting. The large differences between social role performance of individual patients seem to indicate that patients have limited energy and are forced to choose on what role they spend their limited energy.

Interestingly, even though only small differences in empowerment scores were seen between the QFS patients in our sample, those with higher empowerment scores had significantly lower odds of performing less time on almost all social roles, except for parenting and informal care giving. These results underline the importance of empowerment, but also the different nature of specific social roles. Due to the cross-sectional nature of our study, we cannot identify whether empowerment affects social role fulfilment, or the other way around. Nevertheless, this is the first study examining this relation in QFS patients and the results appear to indicate that supporting patients’ empowerment might improve their social functioning. In other chronic diseases, it has been shown that higher levels of empowerment were associated with higher levels of social functioning and work participation, as well as with health status and health-related quality of life [19–22]]. Individual interventions were found more effective in empowering patients and lead to prolonged positive outcomes, compared to group-directed interventions [19, 36]. Especially improvement of knowledge about the chronic disease is associated with increased levels of self-care and self-management [19, 37]. In addition, it can lead to enhanced levels of trust and motivation in chronically ill patients [19, 36]. As interventions to empower patients are available, applying the concept of empowerment to the care and support for QFS patients might be valuable. Future research is needed to explore the impact of empowerment on the lives of QFS patients. A longitudinal design is preferable to gain more insight into the direction of the association and to explore whether interventions focussed on empowerment help QFS patients in their daily functioning.

### Strengths and limitations

To the best of our knowledge, this is the first study in QFS patients focusing on work participation, social roles, and empowerment ≥10 years after Q-fever, providing novel and important insights into these topics. However, this study has some limitations, including the cross-sectional design which prevents us from investigating causalities. Another limitation is restricting the recruitment of QFS patients to only those registered at Q-support. Even though this is the expertise and support centre in the Netherlands, not all QFS patients are registered in the Q-support QFS registry, and registered patients might not be representative of all QFS patients. For example, the proportion of patients with coexisting chronic disease appears to be slightly higher than in the Dutch general population [38].

Besides, response bias might be present as patients experiencing limited impact of QFS might have been less willing to participate. In contrast, patients with severe QFS might also have chosen not to participate due to the time and energy needed to fill in the survey. Furthermore, due to the long-follow up period, there is a risk of recall bias in our results regarding the situation before Q-fever [39]. Regarding the impact of QFS on social roles, several other factors could also have influenced patients’ answers, included the governmental measures taken in response to the COVID-19 pandemic and the changing family situation during the past 10 years.

Also, some methodological decisions might have impacted our results. In case year of Q-fever was unknown, we used the year of the QFS diagnosis to calculate participant’s age at infection to apply our inclusion criteria. As the QFS diagnosis usually takes place year(s) after Q-fever, this might be considered a limitation at first sight. However, the 13 participants for whom this method was applied were all ≥45 years old at time of study, which assured us that they were ≥18 years old at time of infection. Also, we used a shortened version of the Netherlands Empowerment List, which is an extensive questionnaire [16]. Items that were considered most relevant to QFS patients were selected to minimise patient burden and be able to examine a wide range of outcomes of QFS patients. Lastly, the number of determinants included in this study was limited, and other patient characteristics, such as smoking history and other lifestyle factors, should also be included in future research [14].

## Conclusion

More than 10 years after Q-fever, work participation and fulfilment of social roles is generally low in QFS patients. The vast majority of the participants stopped working or works less hours due to QFS, many patients are (partially) incapacitated due to QFS, and the impact on social roles is high. Although only minor variation was present in empowerment scores among participants, higher scores were associated with fulfilment of social roles, but not work participation. This new insight should be examined further in future studies. Healthcare professionals might support empowerment, possibly by offering empowerment interventions, to increase social role fulfilment of QFS patients.

## Supporting information

S1 Fig Flowchart of study sample. (DOCX)

S1 Table Characteristics of subgroups based on pre-infection employment type. (DOCX)

S2 Table Univariate logistic regression analyses for performing a specific social role less than before Q-fever for the roles: Relationship with partner, household activities and parenting. (DOCX)

S3 Table Univariate logistic regression analyses for performing a specific social role less than before Q-fever for the roles: Hobbies, sports and social contacts. (DOCX)

S4 Table Univariate logistic regression analyses for performing a specific social role less than before Q-fever for the roles: Volunteer work, travelling and informal care provision. (DOCX)
