# Supplementary figures and images for: Work participation, social roles, and empowerment of Q-fever fatigue syndrome patients ≥10 years after infection
Source: PLoS One. 2024 Apr 30;19(4):e0302573. doi: 10.1371/journal.pone.0302573 (PMC11060533; doi:10.1371/journal.pone.0302573)

**S1 Figure.** Flowchart of study sample


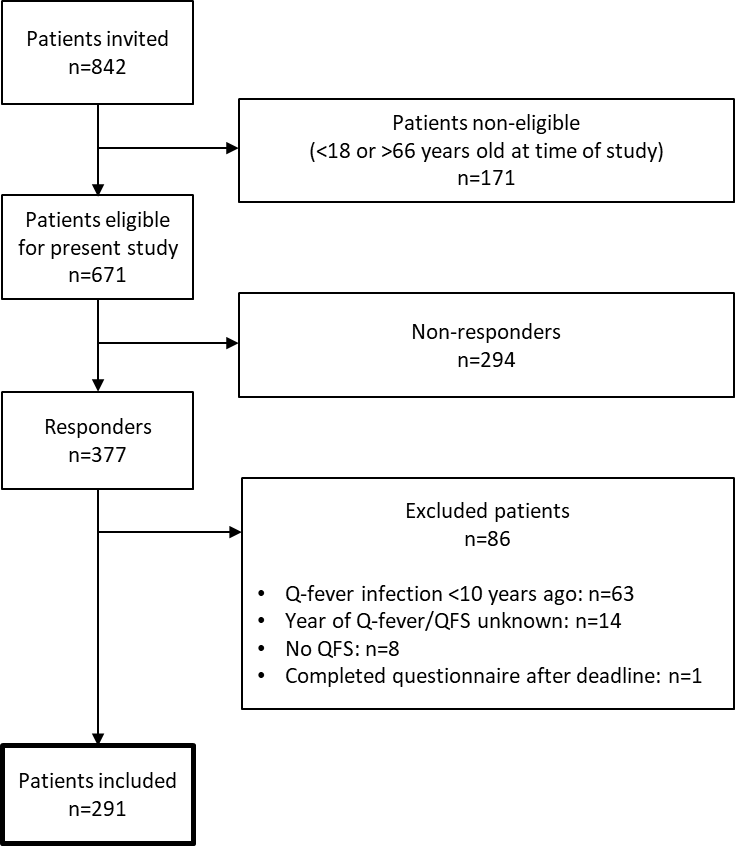

Supplement: S1 Fig — (DOCX) [file pone.0302573.s001.docx]
