# Supplementary material for: Work participation, social roles, and empowerment of Q-fever fatigue syndrome patients ≥10 years after infection
Source: PLoS One. 2024 Apr 30;19(4):e0302573. doi: 10.1371/journal.pone.0302573 (PMC11060533; doi:10.1371/journal.pone.0302573)
Supplement: S1 Table — (DOCX) [file pone.0302573.s002.docx]

**S1 Table.** Characteristics of subgroups based on pre-infection employment type*

^*^Selection of participants not retired at time of filling in the survey (n=290).
**Note**. p-values printed in bold indicate statistically significant values (p<0.05).

|  | | Wage-employed | Self-employed | No employment | P-value for difference |
| --- | --- | --- | --- | --- | --- |
|  | | N=215 | N=35 | N=40 |  |
| Age at time of study*,* median (IQR) | | 54.0 (47.0-60.0) | 59.0 (55.0-61.0) | 53.0 (48.3-61.8) | **0.008** |
| Gender | |  |  |  | **0.002** |
|  | Male | 100 (46.5%) | 23 (65.7%) | 10 (25.0%) |  |
|  | Female | 115 (53.5%) | 12 (34.3%) | 30 (75.0%) |  |
| Level of education | |  |  |  | **0.025** |
|  | Low | 52 (24.2%) | 7 (20.0%) | 17 (42.5%) |  |
|  | Middle | 91 (42.3%) | 19 (54.3%) | 18 (45.0%) |  |
|  | High | 72 (33.5%) | 9 (25.7%) | 5 (12.5%) |  |
| Household composition | |  |  |  | 0.480 |
|  | Married or living with partner, with or without children at home | 156 (72.6%) | 27 (77.1%) | 26 (65.0%) |  |
|  | Living alone or one-parent household with or without children living at home | 59 (27.4%) | 8 (22.9%) | 14 (35.0%) |  |
| Comorbidity | |  |  |  | **0.017** |
| No coexisting chronic disease | | 90 (41.9%) | 10 (28.6%) | 8 (20.0%) |  |
| ≥1 coexisting chronic disease | | 125 (58.1%) | 25 (71.4%) | 32 (80.0%) |  |
| Years since Q-fever infection*,* median (IQR) | | 12.0 (12.0-13.0) | 12.0 (12.0-13.0) | 12.0 (12.0-13.0) | 0.900 |
|  | Before 2007 | 16 (7.4%) | 0 (0.0%) | 1 (2.5%) |  |
|  | Between 2007-2011 | 199 (92.6%) | 35 (100.0%) | 39 (97.5%) |  |
| Antibiotics during the acute phase of the infection | |  |  |  | 0.923 |
| Yes | | 142 (66.0%) | 21 (60.0%) | 26 (65.0%) |  |
| No | | 60 (27.9%) | 12 (34.3%) | 11 (27.5%) |  |
| Not sure | | 13 (6.0%) | 2 (5.7%) | 3 (7.5%) |  |
| Hospitalization during the acute phase of the infection | |  |  |  | 0.360 |
| Yes | | 35 (16.3%) | 9 (25.7%) | 6 (15.0%) |  |
| No | | 180 (83.7%) | 26 (74.3%) | 34 (85.0%) |  |
