# Supplementary material for: Work participation, social roles, and empowerment of Q-fever fatigue syndrome patients ≥10 years after infection
Source: PLoS One. 2024 Apr 30;19(4):e0302573. doi: 10.1371/journal.pone.0302573 (PMC11060533; doi:10.1371/journal.pone.0302573)
Supplement: S2 Table — (DOCX) [file pone.0302573.s003.docx]

**S1 Table.** Univariate logistic regression analyses for performing a specific social role less than before Q-fever for the roles: relationship with partner, household activities and parenting

**Note**. This table presents the odds ratio (OR) for performing a specific role **less** than before Q-fever.

|  | Relationship with  partner | | | Household  activities | | | Parenting | | |
| --- | --- | --- | --- | --- | --- | --- | --- | --- | --- |
|  | N=233 | | | N=273 | | | N=143 | | |
|  | *OR* | *95% CI* | *p-value* | *OR* | *95% CI* | *p-value* | *OR* | *95% CI* | *p-value* |
| Gender |  |  |  |  |  |  |  |  |  |
| Male | 0.656 | 0.381-1.130 | 0.129 | 0.210 | 0.104-0.423 | **<0.001** | 0.782 | 0.401-1.525 | 0.471 |
| Female (ref) |  |  |  |  |  |  |  |  |  |
| Age (continuous) | 0.988 | 0.959-1.017 | 0.410 | 0.985 | 0.953-1.109 | 0.380 | 1.006 | 0.976-1.045 | 0.780 |
| Level of education |  |  |  |  |  |  |  |  |  |
| Low | 0.902 | 0.490-1.661 | 0.740 | 1.977 | 0.878-4.450 | **0.100** | 0.881 | 0.409-1.896 | 0.746 |
| Middle (ref) |  |  |  |  |  |  |  |  |  |
| High | 1.133 | 0.622-2.066 | 0.683 | 1.081 | 0.547-2.136 | 0.823 | 1.072 | 0.506-2.271 | 0.856 |
| Married/living with partner |  |  |  |  |  |  |  |  |  |
| Yes (ref) |  |  |  |  |  |  |  |  |  |
| No | 4.075 | 1.642-10.116 | **0.002** | 1.275 | 0.626-2.595 | 0.503 | 1.486 | 0.631-3.496 | 0.365 |
| Paid work before Q-fever |  |  |  |  |  |  |  |  |  |
| Yes (ref) |  |  |  |  |  |  |  |  |  |
| No | 1.276 | 0.532-3.059 | 0.585 | 1.053 | 0.411-2.697 | 0.914 | 0.508 | 0.191-1.352 | 0.175 |
| Comorbidity |  |  |  |  |  |  |  |  |  |
| None | 0.746 | 0.431-1.291 | 0.295 | 0.872 | 0.466-1.634 | 0.670 | 1.108 | 0.568-2.161 | 0.764 |
| ≥1 (ref) |  |  |  |  |  |  |  |  |  |
| Hospitalization |  |  |  |  |  |  |  |  |  |
| No (ref) |  |  |  |  |  |  |  |  |  |
| Yes | 1.325 | 0.636-2.764 | 0.453 | 2.027 | 0.758-5.424 | 0.159 | 1.126 | 0.447-2.833 | 0.801 |
| Empowerment sum score (continuous) | 0.900 | 0.850-0.954 | **<0.001** | 0.925 | 0.868-0.985 | **0.015** | 0.978 | 0.922-1.038 | 0.460 |
