# Supplementary material for: Work participation, social roles, and empowerment of Q-fever fatigue syndrome patients ≥10 years after infection
Source: PLoS One. 2024 Apr 30;19(4):e0302573. doi: 10.1371/journal.pone.0302573 (PMC11060533; doi:10.1371/journal.pone.0302573)
Supplement: S3 Table — (DOCX) [file pone.0302573.s004.docx]

**S2 Table.** Univariate logistic regression analyses for performing a specific social role less than before Q-fever for the roles: hobbies, sports and social contacts

**Note**. This table presents the odds ratio (OR) for performing a specific role **less** than before Q-fever.

|  | Hobbies | | | Sports | | | Social contacts | | |
| --- | --- | --- | --- | --- | --- | --- | --- | --- | --- |
|  | N=261 | | | N=246 | | | N=266 | | |
|  | *OR* | *95% CI* | *p-value* | *OR* | *95% CI* | *p-value* | *OR* | *95% CI* | *p-value* |
| Gender |  |  |  |  |  |  |  |  |  |
| Male | 0.633 | 0.306-1.308 | 0.217 | 1.148 | 0.532-2.480 | 0.725 | 0.775 | 0.393-1.530 | 0.463 |
| Female (ref) |  |  |  |  |  |  |  |  |  |
| Age (continuous) | 0.973 | 0.934-1.014 | 0.192 | 1.019 | 0.981-1.059 | 0.325 | 0.974 | 0.937-1.012 | 0.171 |
| Level of education |  |  |  |  |  |  |  |  |  |
| Low | 1.991 | 0.736-5.383 | 0.175 | 1.333 | 0.518-3.434 | 0.551 | 1.299 | 0.565-2.989 | 0.538 |
| Middle (ref) |  |  |  |  |  |  |  |  |  |
| High | 0.561 | 0.267-1.178 | 0.127 | 0.604 | 0.275-1.328 | 0.209 | 1.112 | 0.524-2.359 | 0.783 |
| Married/living with partner |  |  |  |  |  |  |  |  |  |
| Yes (ref) |  |  |  |  |  |  |  |  |  |
| No | 1.548 | 0.642-3.730 | 0.331 | 1.353 | 0.553-3.313 | 0.508 | 1.955 | 0.823-4.647 | 0.129 |
| Paid work before Q-fever |  |  |  |  |  |  |  |  |  |
| Yes (ref) |  |  |  |  |  |  |  |  |  |
| No | 1.055 | 0.346-3.221 | 0.925 | 2.000 | 0.451-8.875 | 0.362 | 0.681 | 0.260-1.785 | 0.434 |
| Comorbidity |  |  |  |  |  |  |  |  |  |
| None | 0.855 | 0.410-1.782 | 0.676 | 0.672 | 0.312-1.450 | 0.311 | 0.860 | 0.431-1.720 | 0.671 |
| ≥1 (ref) |  |  |  |  |  |  |  |  |  |
| Hospitalization |  |  |  |  |  |  |  |  |  |
| No (ref) |  |  |  |  |  |  |  |  |  |
| Yes | 0.938 | 0.364-2.421 | 0.895 | 1.300 | 0.428-3.952 | 0.644 | 0.755 | 0.321-1.771 | 0.518 |
| Empowerment sum score (continuous) | 0.871 | 0.803-0.944 | **<0.001** | 0.888 | 0.817-0.964 | **0.005** | 0.904 | 0.841-0.971 | 0.006 |
