# Supplementary material for: Work participation, social roles, and empowerment of Q-fever fatigue syndrome patients ≥10 years after infection
Source: PLoS One. 2024 Apr 30;19(4):e0302573. doi: 10.1371/journal.pone.0302573 (PMC11060533; doi:10.1371/journal.pone.0302573)
Supplement: S4 Table — (DOCX) [file pone.0302573.s005.docx]

**S3 Table.** Univariate logistic regression analyses for performing a specific social role less than before Q-fever for the roles: volunteer work, travelling and informal care provision

**Note**. This table presents the odds ratio (OR) for performing a specific role **less** than before Q-fever.

|  | Volunteer work | | | Travelling | | | Informal care provision | | |
| --- | --- | --- | --- | --- | --- | --- | --- | --- | --- |
|  | N=139 | | | N=228 | | | N=101 | | |
|  | *OR* | *95% CI* | *p-value* | *OR* | *95% CI* | *p-value* | *OR* | *95% CI* | *p-value* |
| Gender |  |  |  |  |  |  |  |  |  |
| Male | 0.265 | 0.116-0.606 | **0.002** | 0.906 | 0.504-1.629 | 0.741 | 0.377 | 0.158-0.900 | **0.028** |
| Female (ref) |  |  |  |  |  |  |  |  |  |
| Age (continuous) | 0.960 | 0.916-1.005 | **0.081** | 1.016 | 0.986-1.048 | 0.289 | 1.006 | 0.960-1.054 | 0.799 |
| Level of education |  |  |  |  |  |  |  |  |  |
| Low | 5.165 | 1.155-23.087 | **0.032** | 1.820 | 0.824-4.017 | 0.138 | - |  | - |
| Middle (ref) |  |  |  |  |  |  |  |  |  |
| High | 0.643 | 0.297-1.395 | 0.264 | 0.927 | 0.495-1.735 | 0.813 | 0.479 | 0.196-1.170 | 0.106 |
| Married/living with partner |  |  |  |  |  |  |  |  |  |
| Yes (ref) |  |  |  |  |  |  |  |  |  |
| No | 3.175 | 1.213-8.307 | **0.019** | 2.761 | 1.268-6.013 | **0.011** | 2.026 | 0.676-5.353 | 0.154 |
| Paid work before Q-fever |  |  |  |  |  |  |  |  |  |
| Yes (ref) |  |  |  |  |  |  |  |  |  |
| No | 0.926 | 0.232-3.700 | 0.914 | 1.107 | 0.418-2.934 | 0.837 | 3.000 | 0.629-14.304 | 0.168 |
| Comorbidity |  |  |  |  |  |  |  |  |  |
| None | 0.818 | 0.377-1.775 | 0.612 | 0.635 | 0.351-1.149 | 0.133 | 0.593 | 0.247-1.427 | 0.244 |
| ≥1 (ref) |  |  |  |  |  |  |  |  |  |
| Hospitalization |  |  |  |  |  |  |  |  |  |
| No (ref) |  |  |  |  |  |  |  |  |  |
| Yes | 1.402 | 0.482-4.079 | 0.535 | 0.753 | 0.353-1.606 | 0.463 | 2.337 | 0.719-7.596 | 0.158 |
| Empowerment sum score (continuous) | 0.884 | 0.814-0.960 | **0.003** | 0.932 | 0.879-0.989 | **0.020** | 0.925 | 0.849-1.007 | **0.074** |
